# Pharmaceutical and drug delivery applications of chitosan biopolymer and its modified nanocomposite: A review

**DOI:** 10.1016/j.heliyon.2022.e10196

**Published:** 2022-08-15

**Authors:** Welela Meka Kedir, Gamachu Fikadu Abdi, Meta Mamo Goro, Leta Deressa Tolesa

**Affiliations:** Department of Chemistry, College of Natural and Computational Sciences, Mettu University, Mettu, Ethiopia

**Keywords:** Chitosan, Biopolymer, Nanocomposite, Pharmaceutical, Drug delivery

## Abstract

Due to their improved structural and functional properties as well as biocompatibility, biodegradability, and nontoxicity, chitosan and its nanoparticles are currently grasping the interest of researchers. Although numerous attempts have been made to apply chitosan and its derivatives to biological applications, few have reported in achieving its pharmacological and drug delivery. The goal of the current work is to provide a summary of the chitosan biopolymer's physical, chemical, and biological properties as well as its synthesis of nanoparticles and characterization of its modified nanocomposites. The drug delivery method and pharmaceutical applications of chitosan biopolymer and its modified nanocomposites are examined in further detail in this research. We will introduce also about the most current publications in this field of study as well as its recent expansion.

## 1 Introduction

Due to their great biocompatibility and biodegradability, biopolymers are crucial in biomedical applications [1, 2]. Biopolymer-based nanoparticles are the most promising nano carriers for delivering various therapeutic drugs to tumor cells such ovarian cancer cell lines because they have good biodegradation and biodistribution in biological systems [3, 4]. Additionally, biopolymers are utilized in a number of biomedical applications, including genes delivery [1], tissue engineering, and drug delivery [5, 6]. Due to their usage and limitations, biopolymers are a major chemistry and biology interface [7, 8]. There is a need to create awareness and create novel ways for biomedical and agricultural applications given the variety of biopolymer applications [1, 9]. The research community is currently very interested in biopolymers such chitosan, alginate, pectin, cellulose, agarose, and gelatin [10, 11].

**Figure 1:**
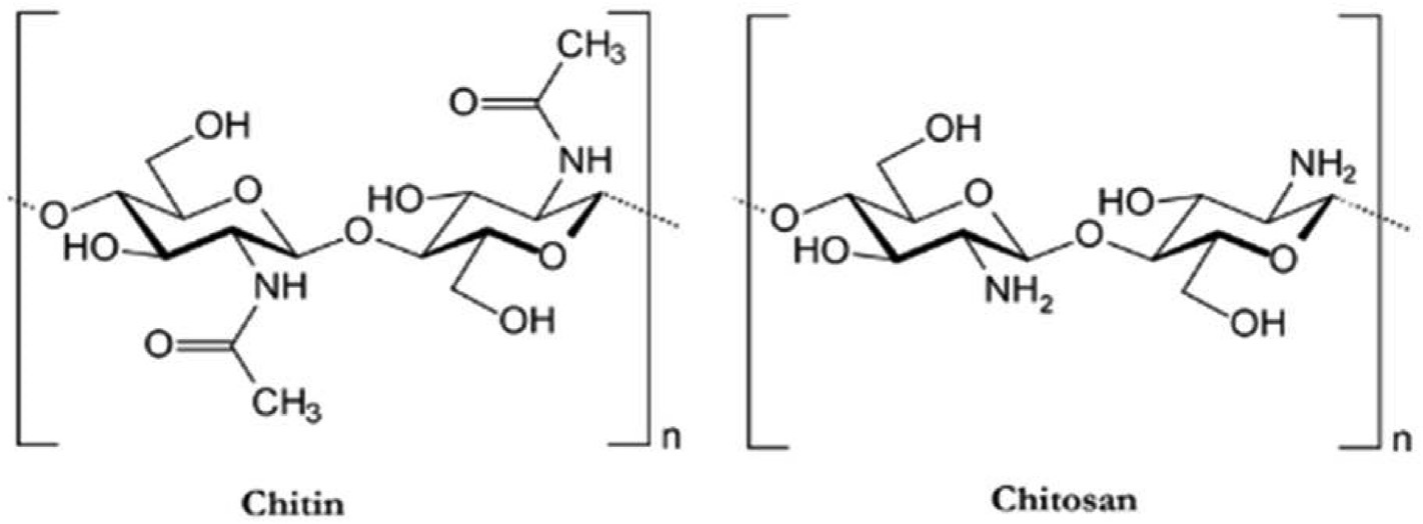
Chemical structure of chitin and chitosan [26].

Chitosan (CS), a biopolymer, has drawn a lot of interest due to its adaptability, accessibility, and special qualities in medical applications [12, 13]. It is made up of 2-acetamido-2-deoxy-D-glucopyranose and 2-amino-2-deoxy-D-glucopyranose units and is the second most prevalent copolymer after cellulose [14, 15, 16]. It is often created from chitin by partial deacetylation in an alkaline environment, as shown structurally in Figure 1 [17-20]. The polymer is digested by human enzymes and helps with wound healing by promoting hemostasis and accelerating tissue regeneration [21, 22, 23]. Additionally, chitosan is made from renewable resources, which are currently extending the range of applications [24]. It has been coupled with a range of polymeric biomaterials and inorganic bioactive chemicals for possible use in orthopedics as bone graft substitutes, intervertebral discs, and bone and cartilage tissue engineering [25].

Due to its antibacterial action, chitosan and its nanoparticles (NPs) are beneficial for a range of biological applications, including food preservation [27]. They also affect fish and crustaceans in an immunomodulatory manner, which directly benefits the aquaculture and fish farming industries [28]. Additionally, CS NPs are now being used to treat illnesses in fish and other animals [29]. Chitosan NPs are attractive candidates for a variety of uses in fish medicine due to their many beneficial biological features, including safety, biocompatibility, biodegradability, and antibacterial capacity [30].

**Figure 2:**
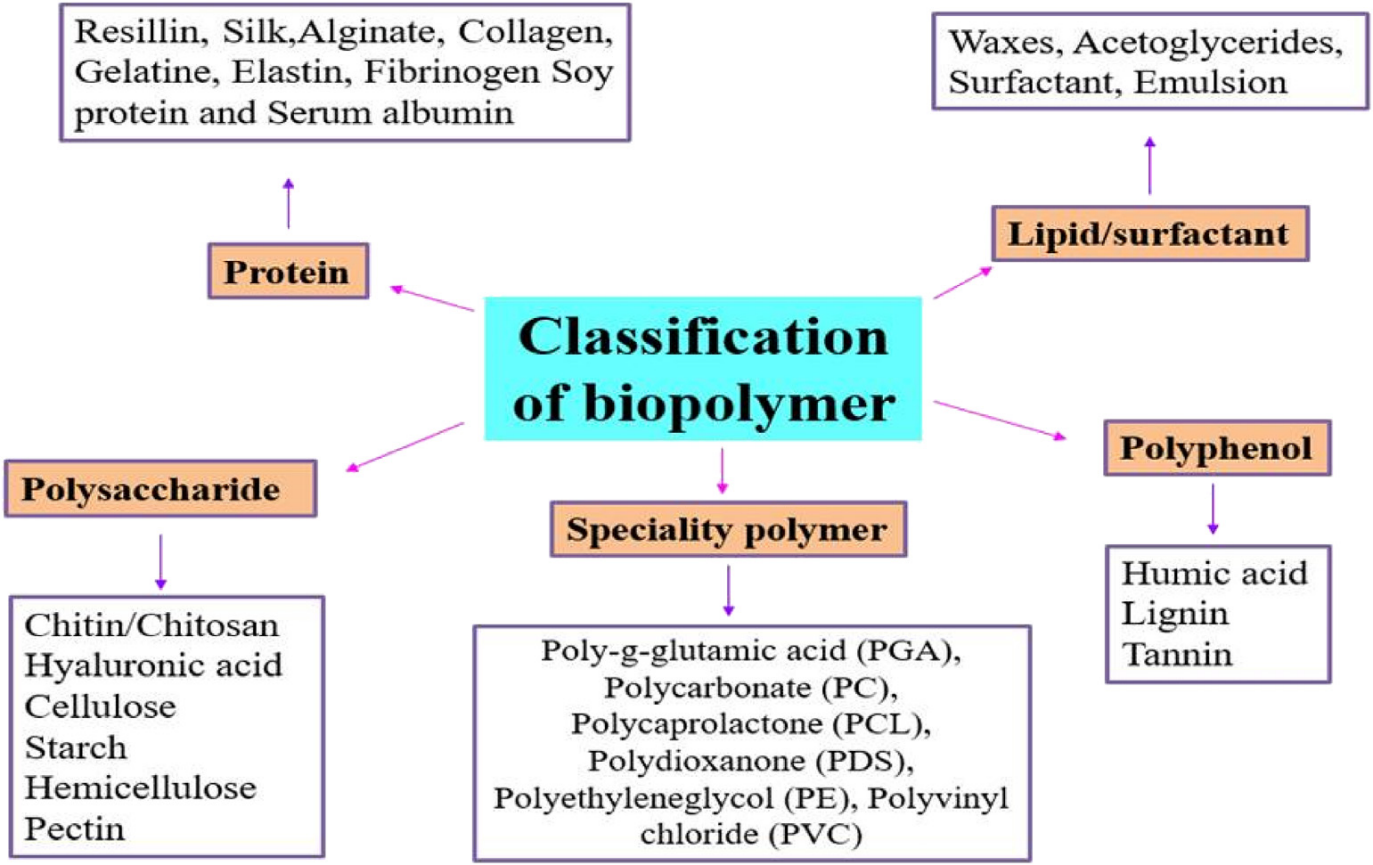
Classification of biopolymers.

Due to its polyelectrolytic nature and its capacity to chelate substances because of the presence of amino groups, chitosan is used for the majority of applications [31, 32]. As a result of its beneficial physicochemical features, which enable the creation of reactive surfaces, chitosan and its derivatives are the materials that are most heavily investigated [33, 34]. Chitosan has numerous uses as a bio-pesticide in agriculture, a packaging material for the food and pharmaceutical sectors, and a membrane filtration system for wastewater treatment [28, 35, 36]. Chitosan is amenable to modification because of the presence of functional groups like amino (NH2+) and hydroxyl (OH-) [37, 38, 39]. Furthermore, chitosan is distinguished by its substantial biological and chemical characteristics and safety [40]. Figure 2 illustrates how distinct groups of biopolymers can be categorized according to the presence and covalent bonding of monomers.

## 2 Physicochemical and biological properties of chitosan biopolymer

Chitosan has a wide range of physicochemical and biological properties, which are listed in Table 1. It can be used in its natural state or in a modified state produced through physical or chemical methods to produce novel qualities and functionalities.

**Table 1:** Summary of the physicochemical and biological properties of chitosan [41, 42].

Physical properties	Chemical properties	Biological properties
• High molecular weight (1.2 × 105 g mol^−1^) • White yellow in color • Weak base (powerful nucleophile, pKa 6.3) • Flakes, bead or powder • Intermolecular hydrogen bonding • Optical clarity. • Amorphous solid • Density 0.18–0.33 g/cm^3^ • Soluble in diluted aqueous acid solution • Insoluble in water • Conductivity	• Rigid D-glucosamine structure • Degree of acetylation range 70–95% • Cationic polyamine • High charge density at pH < 6.5 • Forms gels with Poly-anions Polyelectrolyte • Adheres to negatively charged surfaces • Amiable to chemical modification • Additive in paper industry • Filmogenic properties • Linear polyamine • Numerous reactive groups (amino and hydroxyl) • Linear amino-polysaccharide with high nitrogen content	• Biocompatibility • Bacteriostatic • Wound management • Anticancerogen • Accelerates bone formation • Accelerates the formation of osteoblast • Antioxidant • Biodegradable • Homeostatic • Natural polymer • Bone formation • Safe and non-toxic

## 3 Synthesis of chitosan nanoparticles

Due to their low prices, eco-friendly, and non-toxic natures, metal oxide nanoparticles (NPs) have been created utilizing green chemical technologies in recent decades, and they are good therapeutics for animals and people [43, 44]. Chemically produced metal oxide NPs including hazardous chemical reducing agents such as hydrazinium hydroxide, sodium hypophosphite, and sodium borohydride, on the other hand, have had an environmental impact. The precursors would cling to the broad surfaces of NPs, increasing their toxicity and negatively impacting the environment and biological applications [17, 44, 45]. Chitosan nanoparticles (CNPs) are nontoxic, biocompatible, biodegradable, and functionalized nanostructures derived primarily from by-products of the seafood industry. CNPs have shown potential as green fillers in biodegradable composite reinforcement for food packaging and biomedical applications [44, 46]. In the following section, the majority of the common methods for synthesizing chitosan nanoparticles are thoroughly explained, with their benefits and drawbacks.

### 3.1 Emulsification method

**Figure 3:**
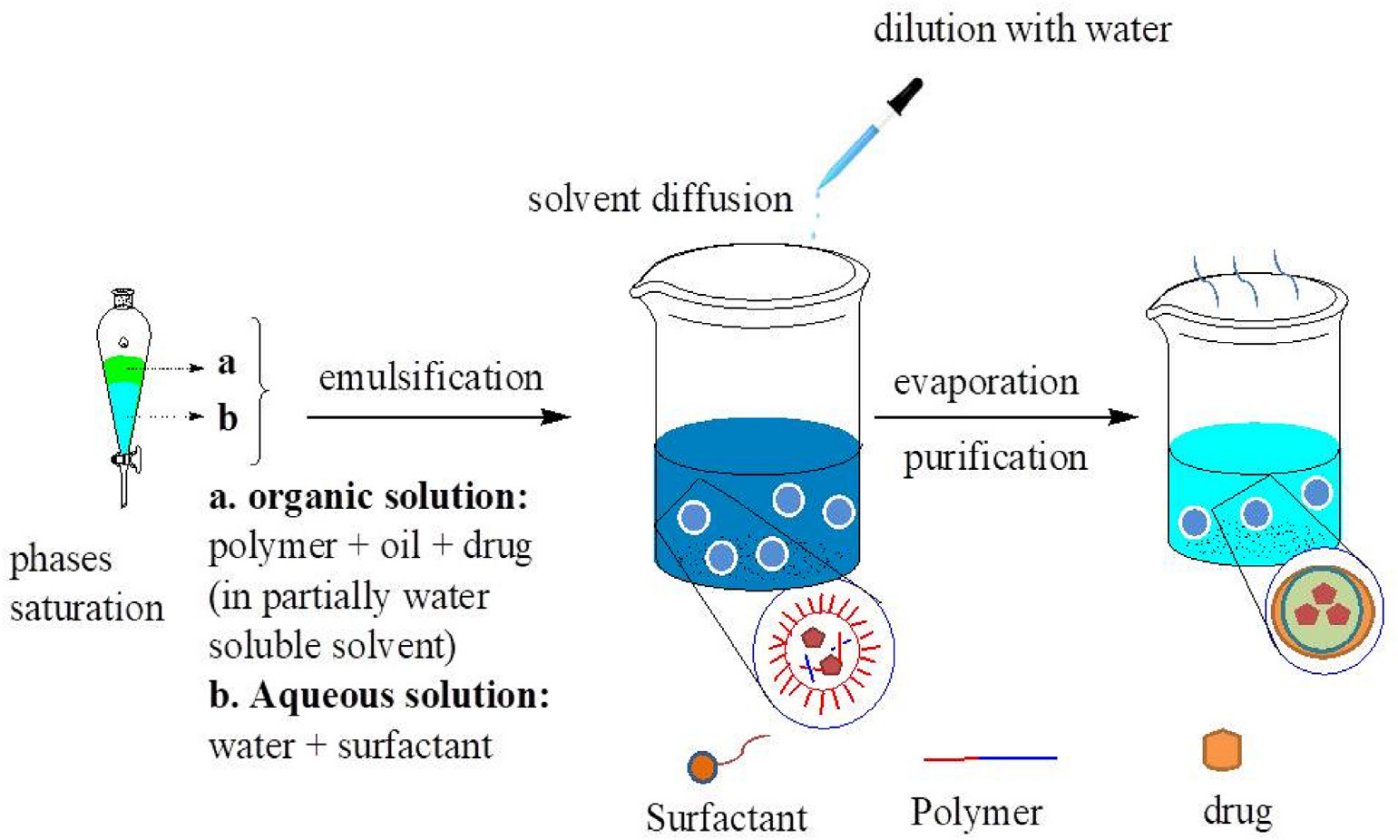
Schematic representation of the emulsification/solvent diffusion method.

**Figure 4:**
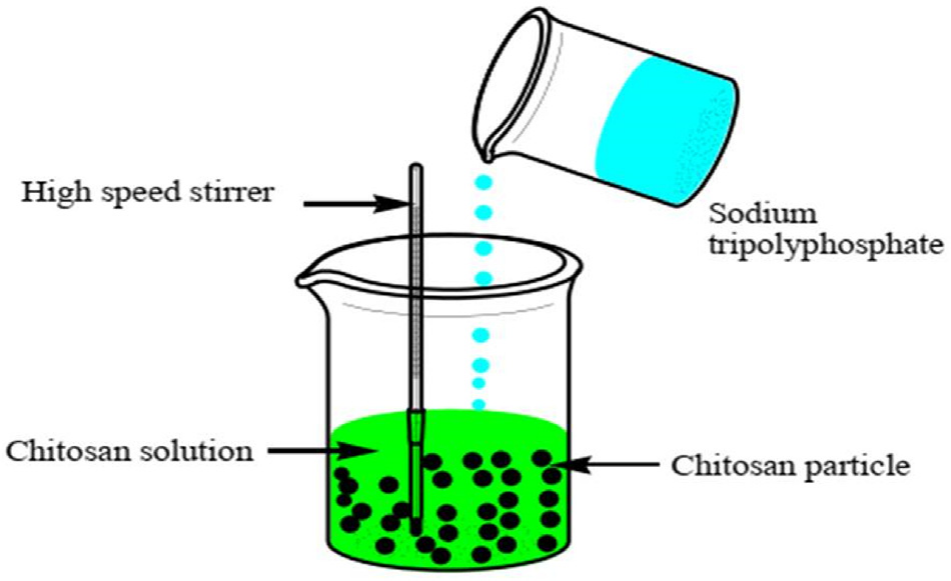
Preparation of chitosan NPs by ion gelation technology.

As depicted in Figure 3 below, emulsions interior phase is made up of a semi-hydrophobic organic solvent like benzyl alcohol or ethyl acetate. Both phases were pre-saturated with water to ensure that they were in thermodynamic equilibrium at ambient temperature [12, 44]. The approach is based on emulsifying a polymer organic solution into a water phase, then evaporating the organic solvent [44, 47]. Following dilution with a large amount of water, solvent diffusion from the dispersed droplets into the outer phase causes the formation of colloidal particles. Finally, evaporation or filtration can be used to remove the organic solvent depending on their boiling point Figure 4 [39,48]. Emulsification reduces the size of the emulsion droplet by using a high-shear force. Following emulsification, the system evaporates the organic solvent under vacuum, resulting in polymer precipitation and the formation of nanoparticles [49]. Finally, NPs with diameters ranging from 80 to 900 nm can be obtained. Despite the need for a large volume of aqueous phase to be removed from the colloidal dispersion and the risk of hydrophilic drug diffusion into the aqueous phase, this method is frequently used for the production of polymeric NPs [39, 50].

### 3.2 Ionic gelation method

Ionic crosslinking is used to create chitosan NPs (Figure 4). A positively charged amine group and a negatively charged polyanion, such as tripolyphosphate (TPP), make up the ionic compound [51, 52]. Chitosan was made into a cationic solution by dissolving it in diluted acetic acid, while TPP was made into an anionic solution by dissolving it in distilled water. The TPP solution was then added one drop at a time to the cationic chitosan solution [48, 53]. At room temperature, mechanical churning created NPs rapidly. Adjusting the amount of chitosan and crosslinking agent, as well as the pH value of the solution, can affect the physiochemical parameters of the resultant NPs, such as particle size and surface charge [54, 55].

### 3.3 Reverse micellar method

**Figure 5:**
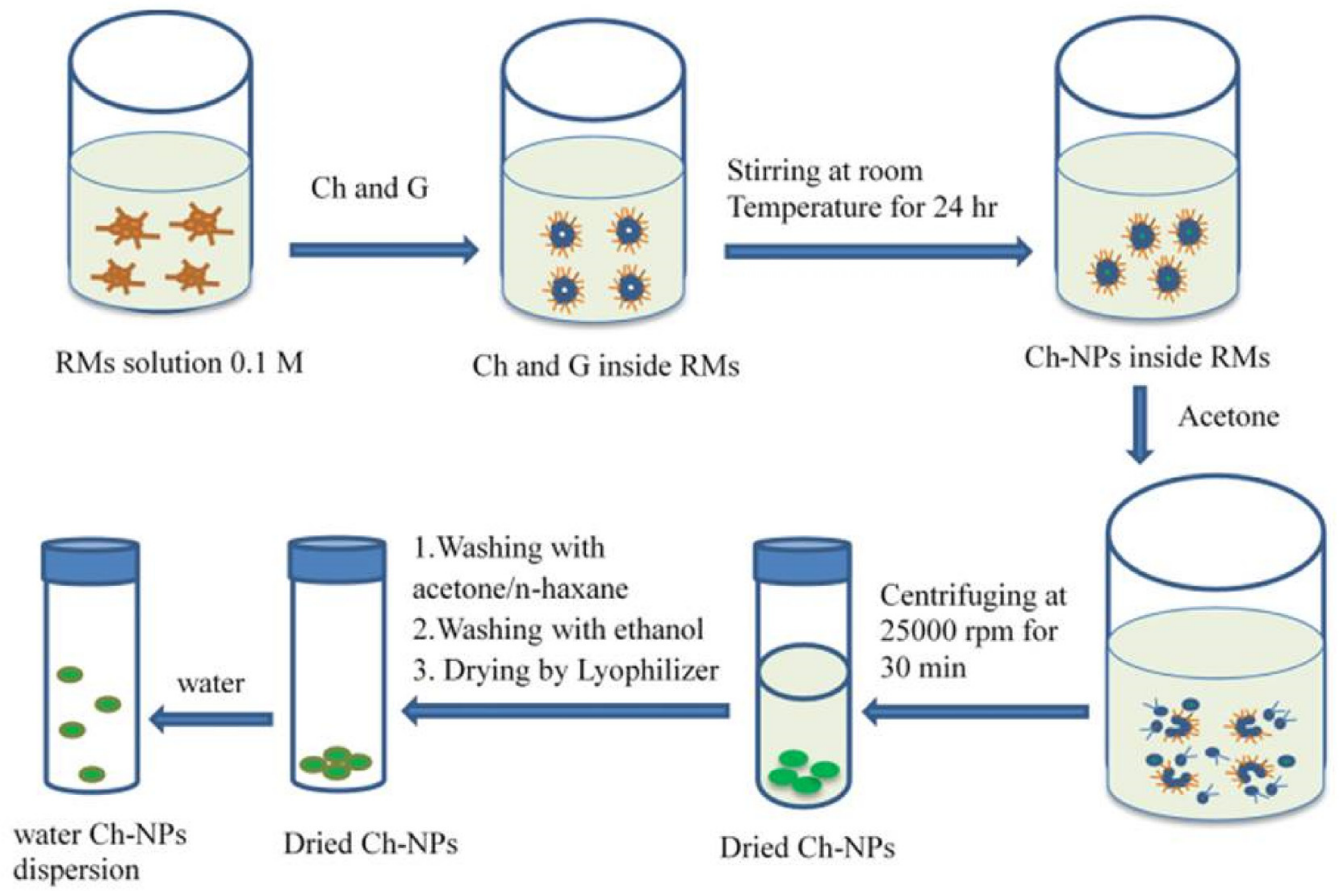
Preparation of chitosan NPs reverses micellar method.

Making chitosan NPs using the reverse micellar technique entails producing NPs in the aqueous core of reverse micellar droplets and then crosslinking them with glutaraldehyde (Figure 5). A surfactant was dissolved in an organic solvent to form reverse micelles in this manner [56]. To prevent turbidity, an aqueous chitosan solution was introduced while constantly whirling [57]. This transparent solution was given a crosslinking agent while being constantly agitated. To complete the cross-linking process and ensure that the free amine group of chitosan was conjugated with glutaraldehyde, the system was kept stirred overnight [56]. The organic solvent was removed by evaporation under low pressure. The surplus surfactant yields and cross-linked chitosan NP yields were achieved. By precipitating the surplus surfactant with an appropriate salt and centrifuging the precipitate, the excess surfactant was removed. The final NPs suspension was dialyzed before lyophilyzation. This approach yielded chitosan NPs with a size of less than 100 nm and a high degree of mono dispersing [48, 56].

### 3.4 Nanoprecipitation method

**Figure 6:**
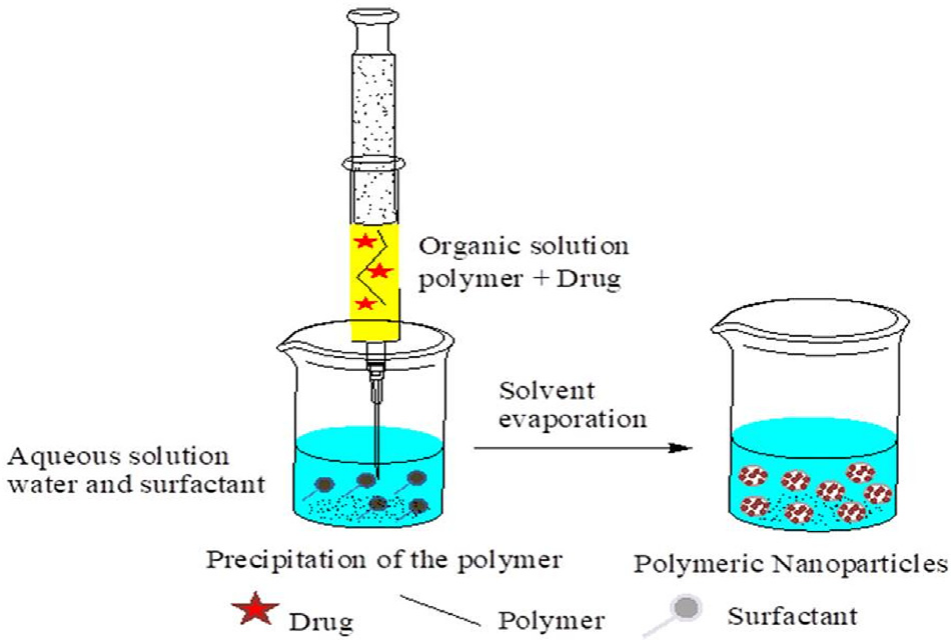
Preparation of chitosan NPs by nanoprecipitation method.

Fessi's group was the first to develop and apply nanoprecipitation, also known as solvent displacement or interfacial deposition [58, 60]. The nanoparticles are made in a colloidal suspension using the nanoprecipitation method, which entails adding the oil phase to the aqueous phase slowly while stirring moderately (Figure 6). It has the advantage of being rapid and simple to utilize because the production of the NPs is instantaneous and takes only one step. The rate of organic phase injection, the rate of aqueous phase agitation, and the oil phase/aqueous phase ratio are all critical manufacturing parameters that have a significant impact on the nanoprecipitation process [50, 59, 61]. Particles with an incredibly narrow dispersion can be created because there is no shearing tension. Entrapment of hydrophobic and hydrophilic medicines is a common application of this method [50, 62]. The polymer and medication are dissolved in a water miscible organic solvent such as acetone or methanol. The solution is then dropped into an aqueous solution containing surfactant one drop at a time. Due to rapid solvent diffusion, the NPs are formed quickly. Following that, the solvents are extracted at a reduced pressure [61, 63].

## 4 Characterization of chitosan biopolymer and its modified nanocomposites

**Figure 7:**
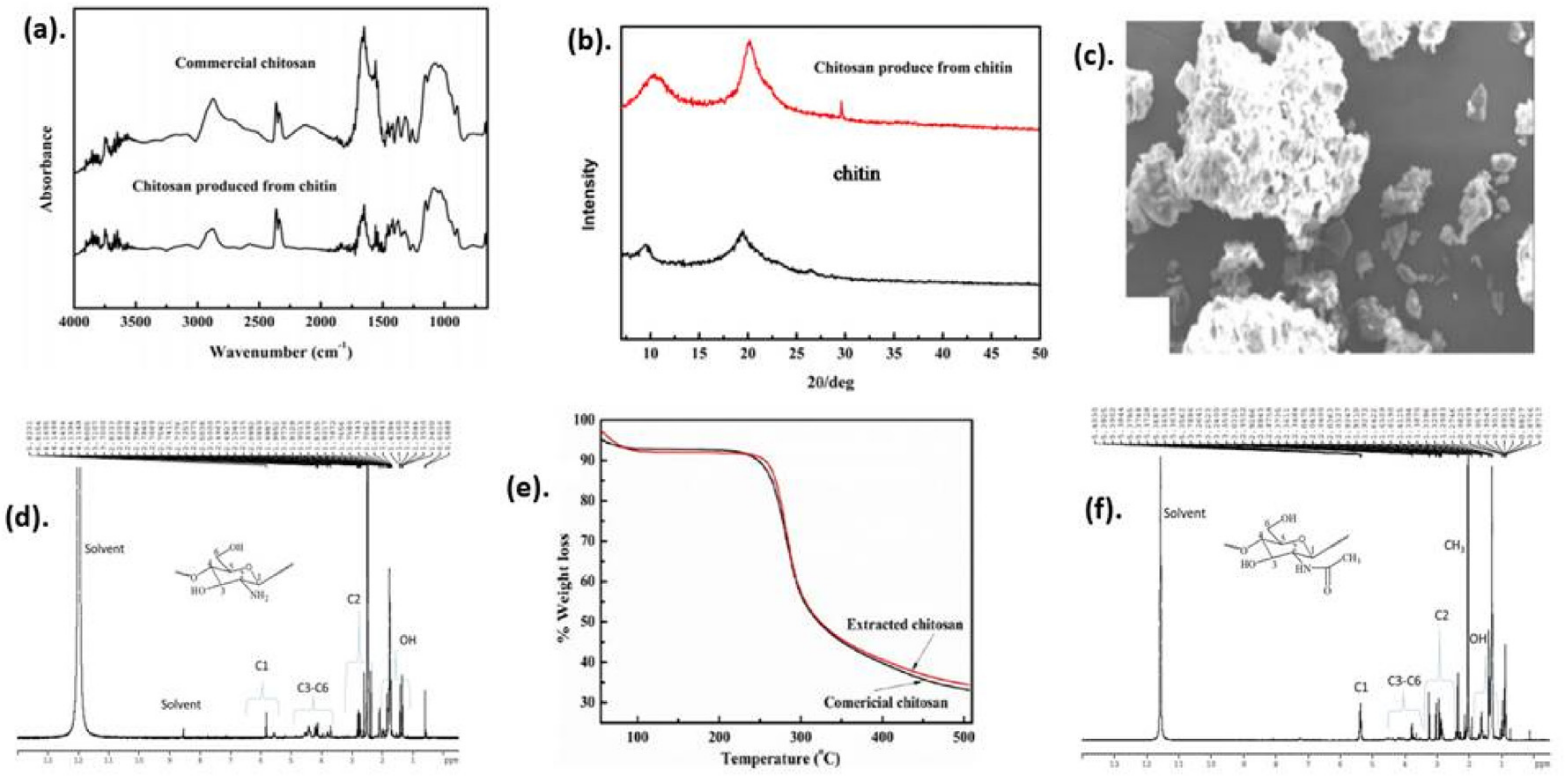
Characterization of chitin and chitosan. (a). FTIR, (b). XRD, (c). SEM, (d and f). ^1^HNMR and (e). Thermogravimetric analysis. Reproduced with permission from [68].

According to the degree of deacetylation (DD), which is assessed by the percentage of D-glucosamine and N-acetyl-D-glucosamine, the biopolymer is classified as either chitin or chitosan [64, 65]. Table 2 shows the most widely used methodologies for characterizing chitosan and its modified nanocomposites. Chromatographic and spectroscopic techniques can be used to analyze chemically modified chitosan derived from chitin, as shown in Figure 7 which is taken from our previous work.

**Table 2:** Instruments commonly used for characterization of chitosan and its modified nanocomposites, as well as their applications.

No	Instruments	Application	Ref
1	Thermogravimetric	Thermal stability of chitosan and its nanocomposites	[66]
2	FT-IR spectroscopy	elucidate the structure of a compound	[67]
3	Viscometric analysis	Molecular weight determination	[68]
4	X-ray diffraction	Crystallinity and phase purity	[69]
5	Scanning electron microscopy	Morphology	[70, 71]
6	^1^H NMR	Characteristic peaks of proton	[71]

## 5 Pharmaceutical applications of chitosan biopolymer, chitosan derivatives and its modified nanocomposites

**Figure 8:**
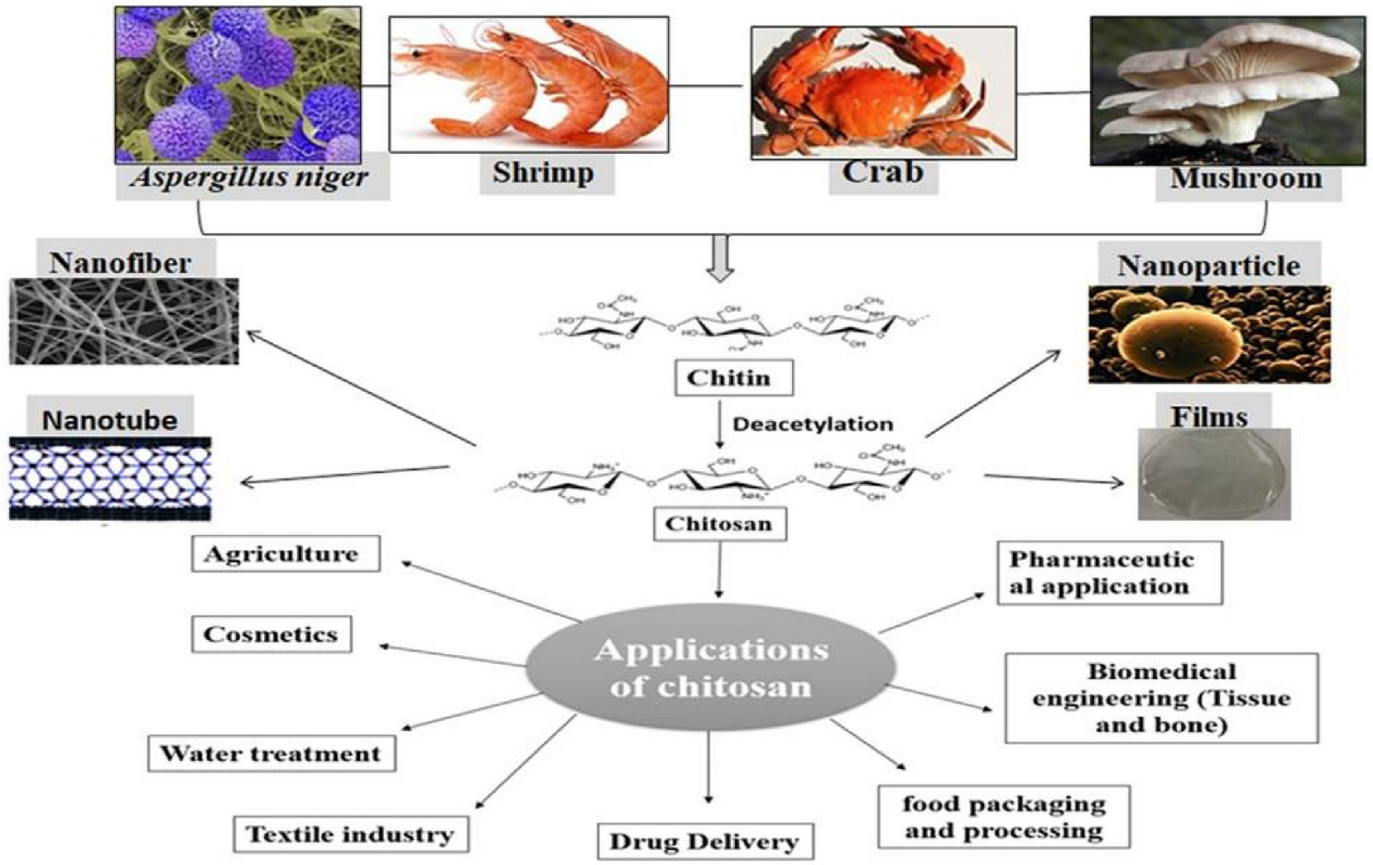
Chitin, chitosan and chitosan nanostructure formation and potential applications.

Chitosan is used in a wide range of sectors, from agriculture to advanced biotechnology and nanotechnology professions (Table 3). Figure 8 shows chitosan derivatives and modified composites applications in wastewater treatment, cosmetics, textiles, biomedical, food packaging and processing, and other applications [72, 73]. Bacterial and viral infections can be life-threatening as a result of drug overuse and the emergence of antibiotic-resistant pathogens. Biopolymers are currently considered to be the most promising medicinal materials [74, 75]. Chitosan is a surface-modified polysaccharide that is used in drug delivery systems [76, 77]. It has attracted a lot of attention because of its biocompatibility, low cost, nontoxicity, environmental friendliness, absorbability, biodegradability, recyclability, and superior antibacterial characteristics [72, 78]. Chemical modifications to the chitosan structure have sparked a lot of attention because they improve transfection efficiency and stability [79, 80].

**Table 3:** Reports chitosan based nanocomposite and their pharmaceutical applications.

Chitosan derivatives and chitosan based nanocomposite	Properties	Pharmaceutical applications	Reference
Chitosan/Polyvinyl alcohol and modified thiabendazolium-montmorillonite.	Biodegradable and antimicrobial activities	Show a good antibacterial activities *against S. aureus* and *E. coli*) and active packaging applications	[30]
Carboxylic acid functionalized carbon nanotubes dispersed in chitosan as a selective layer on the polsulfone membrane (CNTs-COOH/CHIT/PS) Carbon nanotubes dispersed in chitosan as a selective layer on the polsulfone membrane (CNTs/CHIT/PS)	Eco-friendly adsorbent Eco-friendly adsorbent	Efficient rejection of heavy metal ions from aqueous solutions. Less efficient than (CNTs-COOH/CHIT/PS) in rejection of heavy metal ions from aqueous solutions.	[87]
Porous nickel molybdate nanosheets/chitosan (NiMoO_4_/CHIT).	Sensitive, selective, reproducible, biocompatible & biodegradable	As biosensor and practical pharmaceutical analysis (detection of amlodipine drug).	[91]
Gold nanoparticles and a chitosan nanocomposite film coated on a screen printed electrode (Au-NPs/CHI/SPE).	Sensitivity, stability, reproducibility, immuno sensor & cancer biomarkers	Exhibited potential in clinical screening of cancer biomarkers. Diagnosis of prostate cancer using prostate-specific antigen.	[92]
(Nickel Ferrite cores/bovine serum albumin/chitosan/folic acid) NFs-BSA–CS–FA or BSA-CMC-FA conjugates.	Hydrophilicity, nontoxicity, cancer-specific capability and biocompatible	Green approach for breast cancer MR imaging, treatment, tumor diagnosis and therapy.	[93]
Six novel N,N,O tridentate water soluble hydrazide based O-carboxymethyl chitosan Schiff base derivatives	Anti-inflammatory, antioxidant & antidiabetic agent	Could be used for treatment of body pain, as anti-diabetes and cancer.	[94]
Gold and silver-based chitosan nocomposites	antimicrobial, antitumor, anti-inflammatory and antioxidant effects	Possess potential applications in nanomedicine. Used as wound dressing and anti-bacterial activities.	[86]
N,N,N-Trimethyl ammonium chitosan (TMC)	Water solubility, pH sensitivity antibacterial, anti-inflammatory agents	Widely used in medicine as antibacterial, anti-inflammatory drugs, filler fiber in materials for dressing wounds	[95]
Chitosan (CS) Deacetylation Degree(DD) + Alginate (ALG) Chitosan + Lecithin liposomes + L-Arginine	Bioavailability, mucoadhesion and blood glucose lowering properties.	Used as effective insulin oral delivery for treatment of diabetes	[96]
Ag-chitosan nanoparticles	Durable effects and antibacterial activity	Portray encouraging antibacterial reduction of textile materials	[97]
N-quaternized chitosan/poly(vinyl alcohol) hydrogels	Biocompatibility, biodegradability, nontoxicity, availability in abundance and antifungal agent	Used as antifungal agent and in wound dressing materials	[98]
Chitosan beads & Chitosan stabilized bimetallic Fe/Ni nanoparticles, Grafted chitosan hydrogel with acrylic acid, MgO/Chitosan/Graphene oxide and Chitosan-g-poly(glycidyl methacrylate)	Adsorbents of antibiotic pharmaceuticals	Removal of Amoxicillin, Enrofloxacin, Norfloxacin and Cephalosporin respectively from aquatic environment. Used for waste water treatment by removing antibiotic pharmaceuticals.	[99]
2,6-Diamino chitosan (2,6-DAC)	Biodegradable, biocompatible and synergistic activity	Exhibit broad bactericidal efficacy toward both Gram-positive and Gram-negative bacteria with minimum inhibitory concentrations and has synergistic activity with antibiotics including amikacin, tobramycin, novobiocin, rifampicin, and tazobactam.	[100]
Chitosan/polylactic acid/calcium phosphate Chitosan/calcium phosphate nanosheet	Tough bone-resembling and osteoblast enlargement biodegradability and low cytotoxicity	Bone tissue engineering (bone implants) Vaccine carrier	[101]
Carboxymethyl chitosan (CMCs) + glutathione-glycylsarcosine (G-GS) & carboxymethyl chitosan (CMCs) + glutathione-valyl-valin (G-VV)-LDH hybrid	Noncytotoxicity and permeability	As topical administration drug delivery to the posterior segment of the eye.	[102]
Fucoidan-based chitosan carrier	Non toxicity and biocompatibility	Used test Human breast cancer cell line and Colon cancer Caco-2 cells and treatment.	[103]
Chemically modified O-carboxymethyl chitosan Schiff base and their metal complex	Good solubility in water, high viscosity, low toxicity and biocompatibility	Possess better antibacterial, antifungal, anti-inflammatory, antidiabetic and antioxidant	[104]
modified cellulose and cross-linked chitosan with covalently bound 8-hydroxyquinoline	Non-digestible, non resorbable, biocompatible	Potential for treatment of Wilson's disease.	[105]
Chitosan-g-poly (N-isopropyl acrylamide)	Biodegradable and injectable thermo gel, antioxidant and drug delivery	Suppressing oxidative stress, lowering ocular hypertension, reducing retinal ganglion cell loss and enhancing myelin growth and neuron regeneration.	[106]

The -NH_2_ and -OH groups of chitosan molecules make them a good base for interacting with other monomers, biological molecules, polymers, and nanoparticles [20, 81]. A variety of methods can be used to make quality films, fibers, gels, microspheres-microcapsules, and micro/nanoparticles [82, 83]. Because of their physicochemical properties, chitosan and its derivatives are good materials for biomedical and pharmaceutical applications, and they are also compatible with the human body environment [84, 85]. In biological systems, chitosan-based nanoparticles have good biodegradation and bio distribution, making them one of the most promising nano carriers for delivering various therapeutic medicines to tumor cells, particularly ovarian cancer cell lines [4, 79]. In recent years, metal and chitosan composites have been a hotspot of antibacterial research, with the addition of metals to chitosan increasing its antibacterial activity and potentially having applications in nanomedicine [86, 87]. Antibacterial activity of metal-chitosan nanocomposite films was found to be superior to that of chitosan [^88^, ^89^]. Biotechnologists and microbiologists have created various types of chitosan nanocomposites for distinct uses in the biomedical and pharmaceutical industries due to its outstanding physical, chemical, and biological properties [56, 88]. Chitosan biopolymer is a remarkable substance for cosmetics, food, medicine, and pharmacy because of these properties [82]. As a result, a number of researchers in a variety of fields have contributed to the field of chitosan-based nanocomposites, and a variety of chitosan-based materials have been made and evaluated for bioactivity studies [89, 90].

### 5.1 Chitosan biopolymer and its modified nanocomposite for drug delivery system

**Figure 9:**
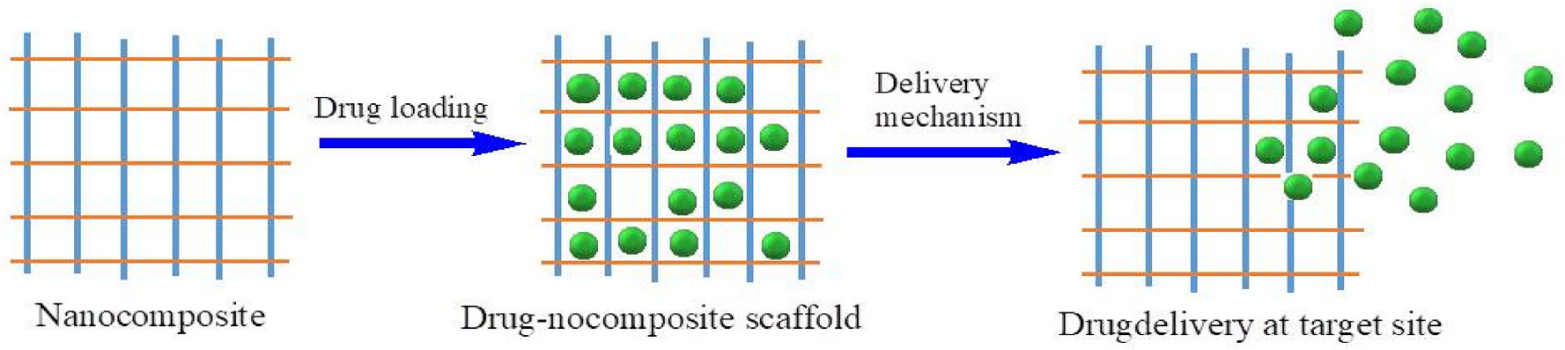
Schematic diagram of drug loading and delivery mechanism by biopolymer nanocomposites.

Natural polymers are considered appropriate hosting materials for nanoparticles, particularly for biological applications, due to their sustainability, eco-friendliness, nontoxicity, biodegradability, and biocompatibility [107, 108]. The development of effective drug delivery techniques that allow bioactive molecules to reach their site of action despite avoiding non-target cells, organs, or tissues is becoming a public health research priority [109, 110]. Drug targeting and regulated drug delivery are concepts that are used to increase the therapeutic index of drugs by improving their localization to specific parts of the human body and reducing potentially detrimental side effects under normal circumstances [111, 112]. This method has a number of benefits, including easy drug adjustments after parenteral administration to achieve target disease sight, increased drug treatment efficacy, and less drug side effects [113, 114]. The medicines can be incorporated into the systems without passing through any chemical processes, which is important for preserving drug activity, and the system can be used for a range of administration routes, such as oral, nasal, parenteral, and intraocular [115, 116]. Figure 9 shows the schematic representation of the drug loading and delivery system for biopolymer nanocomposites.

**Figure 10:**
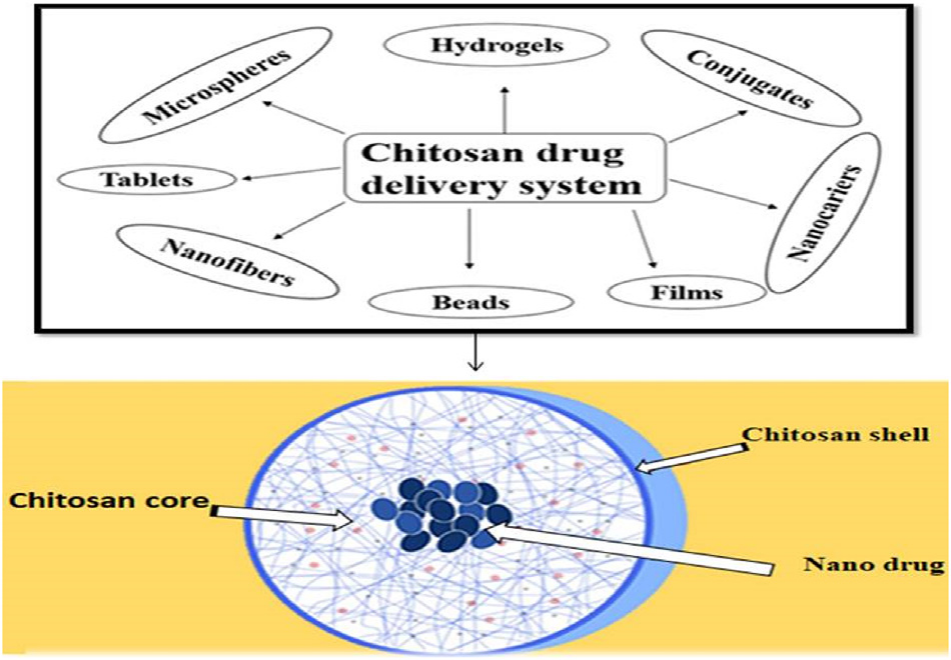
Processing of chitosan and its modified nanocomposite for drug delivery system.

Chitosan nanocomposites, which can be modified for drug delivery system (Figure 10), are frequently employed in the treatment of diseases such as cancer and osteoarthritis [117, 118]. Drug-embedded nanocomposites provide a number of advantages, including improved pharmacokinetics and the capacity to deliver pharmaceuticals to the right location or tumor [119, 120]. A number of recent research have established the capacity to synthesize and describe chitosan biopolymer modified with nano-clay, reduced graphene oxide, zeolites, SiO_2_, hydroxyapatite, and gold nanoparticle for use as a targeted drug carrier in drug delivery systems (Table 4).

**Table 4:** Chitosan biopolymer based drug delivery system with fabricated materials and loaded drugs.

Natural polymer	Method of preparation	Modified material	Drug/model drug	Reference
Chitosan	Spray-drying	-	Diphenylhydamine and mebeverine	[121]
	Ionic gelation	Chitosan -β-cyclodextrin grafted N- maleoyl	Cyclodextrin	[122]
	Nano/microencapsulation methods	Chitosan and Poly(lactide-co-glycolide) (PLGA).	-	[63]
Carboxymethyl chitosan	Hydrothermal method	Carboxymethyl chitosan - folate/Fe3O4/CdTe nanoparticle	Adriamycin	[85]
Chitosan	Precipitated and solvent method	Chitosan -Clay	Ibuprofen	[123]
	ionic-gelation method	Ionically Cross-Linked Chitosan and sodium tripolyphosphate (STPP)	Docetaxel	[52]
	Freeze-drying	Chitosan and Calcium carbonate	Methotrexate	[7]
	Esterification reaction	Folate modified chitosan/carboxymethyl	Paclitaxel	[93]
	Freeze-drying	Catechol modified-Chitosan -Genipin	Sulfasalazine	[7]
N-maleoyl chitosan	Precipitation	N-maleoyl chitosan -β-cyclodextrin	Ketoprofen	[124]
Chitosan	Dissolution	Chitosan and 2-chloro-N,N-diethylethylamine hydrochloride	Quercetin	[19, 125]
	Freeze-drying	Chitosan/Succinic anhydride, glutaric anhydride	Paclitaxel and docetaxel	[122]
	Oxidation	Chitosan/Glycidyltrimethyl ammonium chloride, gelatin	Dopamine	[7]
	Freeze-drying	Chitosan/Poly(DL-lactide-co-glycolide)	Donepezil	[122]
	-	lauryl succinyl/Chitosan/tripolyphosphate	Insulin	[65]
	Crosslinking methods	Chitosan/5-fluorouracil	5-fluorouracil	[62]
	Encapsulation	Chitosan nanoparticles loaded with plasmid DNA encoding Rho1-GTPase protein of Schistosoma mansoni.	-	[126]
	Ionotropic gelation	Chitosan–fluorescein isothiocyanate-bovine serum albumin	fluorescein	[25]
	Freeze-drying	Chitosan/Gold nanoparticle	Curcumin	[127]
Chitosan,aspartate, glutamate, and hydrochloride	Dispersion	AgSD- incorporated bilayer chitosan wound dressing	silver sulfadiazine (AgSD)	[128]
Chitosan	Ionic-gelation method	Chitosan and alginate	Amygdalin	[129]
	Ionic cross-linking	Chitosan and Graphene	Isosfamide	[18]
	Ionic gelation	Chitosan and xanthan gum	Ciprofloxacin	[130]
	Complex coacervation	CS/Dz13Scr NPs	Insulin	[131]
	Ionic cross-linking	Chitosan cross-linked-6-phosphogluconic Trisodium	-	[132]

## 6 Conclusion

Biopolymer nanocomposite have attracted a lot of research due to its special qualities, which include biocompatibility, biodegradability, and nontoxicity as well as better structural and functional features. The most difficult component of this technology is developing bio-based materials with equivalent quality and functions to synthetic materials. Various naturally occurring polymers, such as starch, collagen, alginate, cellulose, and chitin, are appealing candidates because they can reduce reliance on manufactured goods while remaining environmentally beneficial. Chitosan is one of the most exploited biopolymers in biomedical science, and it is the second most abundant next to cellulose, a naturally occurring amino polysaccharide. De-acetylated chitin and its amino-polysaccharide present in nature are used to make chitosan biopolymer. Because of its biocompatible and biodegradable nature, it has inspired a lot of interest in biological applications. Based on various reported study chitosan has been used in a variety of pharmaceutical application including antimicrobial, antioxidant, anti-inflammatory, anticancer and drug delivery systems throughout the last few decades. A range of chitosan sources, modification procedures, and manufacturing methods are also widely discussed. This review stated that chitosan and its nanocomposite have a bright future with improved distinctive qualities of their especial biocompatibility, biodegradability, mechanical and thermal stabilities, barrier, and nontoxicity, suggesting their uniqueness in the biomedical application based on numerous recent publications.

## Declarations

### Author contribution statement

All authors listed have significantly contributed to the development and the writing of this article.

### Funding statement

This research did not receive any specific grant from funding agencies in the public, commercial, or not-for-profit sectors.

### Data availability statement

Data included in article/supplementary material/referenced in article.

### Declaration of interest’s statement

The authors declare no conflict of interest.

### Additional information

No additional information is available for this paper.
